# The Vestibulocerebellum and the Shattered Self: a Resting-State Functional Connectivity Study in Posttraumatic Stress Disorder and Its Dissociative Subtype

**DOI:** 10.1007/s12311-022-01467-4

**Published:** 2022-09-19

**Authors:** Daniela Rabellino, Janine Thome, Maria Densmore, Jean Théberge, Margaret C. McKinnon, Ruth A. Lanius

**Affiliations:** 1 Department of Psychiatry, Western University, University Hospital, (Room C3-103), 339 Windermere Road, London, ON N6A 5A5 Canada; 2 Imaging, Lawson Health Research Institute, London, ON Canada; 3 Department of Psychiatry and Behavioural Neurosciences, McMaster University, Hamilton, ON Canada; 4 Department of Theoretical Neuroscience, Central Institute of Mental Health Mannheim, Medical Faculty Mannheim, Heidelberg University, Heidelberg, Germany; 5 Clinic for Psychiatry and Psychotherapy, Central Institute of Mental Health Mannheim, Medical Faculty Mannheim, Heidelberg University, Heidelberg, Germany; 6 Department of Medical Biophysics, Western University, London, ON Canada; 7 Homewood Research Institute, Guelph, ON Canada; 8 Mood Disorders Program and Anxiety Treatment and Research Centre, St. Joseph’s Healthcare Hamilton, Hamilton, ON Canada; 9 Department of Neuroscience, Western University, London, ON Canada

**Keywords:** Flocculus, Vestibular, PTSD, Dissociation, Functional connectivity, Bodily self-consciousness

## Abstract

The flocculus is a region of the vestibulocerebellum dedicated to the coordination of neck, head, and eye movements for optimal posture, balance, and orienting responses. Despite growing evidence of vestibular and oculomotor impairments in the aftermath of traumatic stress, little is known about the effects of chronic psychological trauma on vestibulocerebellar functioning. Here, we investigated alterations in functional connectivity of the flocculus at rest among individuals with post-traumatic stress disorder (PTSD) and its dissociative subtype (PTSD + DS) as compared to healthy controls. Forty-four healthy controls, 57 PTSD, and 32 PTSD + DS underwent 6-min resting-state MRI scans. Seed-based functional connectivity analyses using the right and left flocculi as seeds were performed. These analyses revealed that, as compared to controls, PTSD and PTSD + DS showed decreased resting-state functional connectivity of the left flocculus with cortical regions involved in bodily self-consciousness, including the temporo-parietal junction, the supramarginal and angular gyri, and the superior parietal lobule. Moreover, as compared to controls, the PTSD + DS group showed decreased functional connectivity of the left flocculus with the medial prefrontal cortex, the precuneus, and the mid/posterior cingulum, key regions of the default mode network. Critically, when comparing PTSD + DS to PTSD, we observed increased functional connectivity of the right flocculus with the right anterior hippocampus, a region affected frequently by early life trauma. Taken together, our findings point toward the crucial role of the flocculus in the neurocircuitry underlying a coherent and embodied self, which can be compromised in PTSD and PTSD + DS.

**Supplementary Information:**

The online version contains supplementary material available at 10.1007/s12311-022-01467-4.

## Introduction

Increasingly, the cerebellum is recognized as contributing to cognitive and affective processes that extend beyond its role in motor performance. Here, the *Universal Cerebellar Transform Hypothesis* postulates that the cerebellum plays a critical role in integrating sensory, limbic, and higher-order cortical inputs, thus maintaining a homeostatic baseline and improving performance in context-dependent behavioral, emotional, and cognitive domains [91]. This theory has been critical in forming a comprehensive understanding of the diverse functions cerebellar regions play in cognition, emotion regulation, action performance, and perception. In the present report, we focus our attention on the floccular lobe and alterations in its functioning among individuals with post-traumatic stress disorder (PTSD) and its dissociative subtype (PTSD + DS).

The flocculus is located in the vestibulocerebellum (hemisphere X of the cerebellum), the most phylogenetically ancient part of the cerebellum. Through its efferent and afferent projections to and from the vestibular complex in the brainstem via the inferior cerebellar peduncle, the flocculus is involved primarily in regulating movement for optimal posture, balance, and the vestibulo-ocular eye reflex [42]. Here, the vestibulocerebellum guides eye, head, and neck movements to compensate for acceleration of the head in a linear or rotational fashion, allowing for the maintenance of a visual target fixed on the retina. The flocculus is also involved in the control of saccades (rapid eye movements occurring between two fixation points), smooth pursuit (SP, slow pursuit of a target in motion), and the optokinetic reflexive eye movements. The optokinetic reflex regulates involuntary eye movements (called nystagmus) in response to motion of the visual field [81], which is crucial for orienting oneself in the environment and for maintaining a constant point of fixation in the visual field.

Functional imaging in humans has highlighted the neural activity of the flocculus during optokinetic reflex, saccadic eye movements, and smooth pursuit [31, 117]. Optokinetic eye movements were traced in a sample of healthy volunteers via functional magnetic resonance imaging (fMRI; [86]), revealing neural activity of the flocculus during horizontal and vertical optokinetic nystagmuses (eye movement elicited by a moving environment in the visual field). Moreover, recent investigation highlights the role of the flocculus in auditory functioning and tinnitus [69], where a feedback loop has been hypothesized between the flocculus (and paraflocculus) and the auditory cortex. Along with the non-motor functions of the cerebellum [89, 92–94], ground-breaking work has revealed structural and functional connectivity of the cerebellum (flocculus included) with limbic regions, specifically with the hippocampus [5, 128], thereby suggesting a role of the cerebellum in emotion regulation and spatial and temporal processing. Finally, higher cortical structures appear functionally connected to the flocculus for eye movement modulation, including the frontal eye field (FEF), the supplementary eye field, the cingulate eye field, the parietal eye field (PEF), the parietal operculum, the middle temporal and intraparietal regions, and visual areas in the occipital cortex [81, 86, 117].

Emerging evidence suggests vestibular and oculomotor impairment in psychological disorders. Here, a recent study revealed decreased functional connectivity between the flocculus, the thalamus, and the FEF in individuals with bipolar disorder as compared to healthy subjects [24], a finding that is in line with previous research suggesting altered saccadic eye movements in uni- and bipolar depression [22]. Similarly, schizophrenia, affective disorders, and obsessive–compulsive disorder have been associated with smooth pursuit deficits [63]. A comprehensive review [40] summarized a range of oculomotor deficits relative to saccadic performance in several psychiatric conditions, including schizophrenia, mood disorders, borderline personality disorder, attention-deficit hyperactivity disorder, and obsessive–compulsive disorder. State and trait anxiety, as well as anxiety disorders, are also thought to impact negatively oculomotor reflexes and gaze [104, 126], with associated dizziness, unsteadiness, and fear of falling. Despite the fact that psychological trauma and chronic stress have been linked to altered functioning in vestibular circuits and performance [45, 87] and abnormal visual functioning, such as blurred vision in veterans [111] and oscillopsia (a visual disturbance where the visual field is perceived in motion; [114]), work examining vestibulo-ocular functioning in trauma-related disorders is scant.

Among trauma-related disorders, PTSD has a relatively high prevalence among the civilian population, with lifetime prevalence rates of 6.8% (lifetime prevalence) in the population of the USA [53] and 9.2% in Canada. PTSD is a psychiatric disorder stemming from experiencing or witnessing a traumatic event, and is characterized by symptoms of hypervigilance, reliving, negative mood and cognitions, and avoidance symptoms [4]. A subgroup of individuals with PTSD (an average of 14% among the PTSD population worldwide; [105]) presents with dissociative symptoms (PTSD + DS), including depersonalization, where the individuals experience detachment from their body or parts of their body, and derealization, where the surroundings are perceived as surreal or dream-like [58, 60, 105, 118]. Previous investigations in PTSD have revealed altered functioning of brain regions involved in vestibular and oculomotor functioning, including for the cerebellum [7, 21, 84], the vestibular circuits—encompassing the vestibular nuclei in the brainstem [45], the insula [36, 46, 50, 74], the temporo-parietal junction [75, 83, 106, 125]—the frontal eye field [84, 88], middle temporal regions [56, 76], and the parietal operculum [120]. Critically, many of these abnormalities are present at rest.

Taken together, functional neuroimaging research and clinical reports suggest a potential association between vestibular and oculomotor impairments and PTSD. Critically, however, no research has been conducted on the neural functioning of the vestibulocerebellum, including the flocculus, in PTSD and its dissociative subtype. We therefore aimed to uncover potential alterations in functional connectivity of the flocculus at rest in PTSD and PTSD + DS as compared to healthy controls. We hypothesized that, even at rest, the vestibulo-ocular circuit in PTSD would reveal specific alterations in its functional connectivity with brainstem, limbic, and cortical areas as compared to controls. In light of previous reports pointing toward a link between vestibular dysfunction and depersonalization/derealization symptoms [52, 66, 127], we also expected dissociative symptomatology in PTSD (PTSD + DS) to distinguish further the functional connectivity of the flocculus as compared to PTSD and healthy controls.

## Methods

### Participants

Participants were recruited via community advertisement and through mental health professionals in the London (ON) region. The final sample included 133 participants, comprising 44 healthy controls, 57 participants with a diagnosis of PTSD, and 32 participants with a diagnosis of PTSD + DS (see the “Methods” section for details on inclusion/exclusion criteria based on functional imaging quality). Part of this sample was previously analyzed in studies from our group investigating functional connectivity of different brain areas at rest [45, 47, 75, 84, 110]. The studies from which these data were drawn were approved by the Research Ethics Board of Western University, and all participants provided informed written consent. Demographic and clinical characteristics of the sample are reported in Table 1.

**Table 1:** Description of the participants’ groups

*Demographic and clinical characteristics*	PTSD group (*n* = 57)	PTSD + DS (*n* = 32)	Control group (*n* = 44)	*p*	Contr vs. PTSD *p*	Contr vs. PTSD + DS *p*	PTSD vs. PTSD + DS *p*
Age (mean ± SD) years	38.32 ± 12.16	41.06 ± 13.09	34.68 ± 12.32	0.122			
Sex (F) frequency	40	28	34	0.178			
CAPS-IV tot (mean ± SD)	66.28 ± 12.27 (*n* = 35)	79.83 ± 13.69 (*n* = 18)	0.136 ± .66 (*n* = 44)	< .0001	< .001	< .001	0.071
CAPS-IV Dep (mean ± SD; range)	0.09 ± 0.51; 3 (*n* = 35)	2.94 ± 2.51; 6 (*n* = 18)	0 (*n* = 44)	< .001			
CAPS-IV Der (mean ± SD; range)	0.31 ± 0.90; 3 (*n* = 35)	3.33 ± 2.70; 8 (*n* = 18)	0 (*n* = 44)	< .001			
CAPS-5 tot (mean ± SD)	36.90 ± 8.79 (*n* = 22)	41.35 ± 8.73 (*n* = 14)	n/a	0.244			
CAPS-5 Dep (mean ± SD); range	0.18 ± 0.50; 2 (*n* = 22)	2.29 ± 1.14; 4 (*n* = 14)	n/a	< .0001			
CAPS-5 Der (mean ± SD); range	0.23 ± 0.61; 2 (*n* = 22)	1.79 ± 0.97; 3 (*n* = 14)	n/a	< .0001			
MDI tot score (mean ± SD)	54.21 ± 24.77 (*n* = 56)	84.9 ± 24.6 (*n* = 31)	34.25 ± 4.18 (*n* = 43)	< .001	< .001	< .0001	< .001
CTQ tot score (mean ± SD)	57.68 ± 23.82 (*n* = 57)	67.74 ± 17.95 (*n* = 30)	31.83 ± 9.20 (*n* = 43)	< .001	< .001	< .001	0.049
BDI tot score (mean ± SD)	23.3 ± 8.56 (*n* = 56)	36.16 ± 13.20 (*n* = 31)	1.16 ± 2.09 (*n* = 43)	< .0001	< .001	< .0001	0.002
Post-scan STAI tot score (mean ± SD)	5.56 ± 1.9 (*n* = 50)	5.9 ± 2.16 (*n* = 29)	3.62 ± 1.27 (*n* = 40)	< .001	< .001	< .001	0.617
Post-scan CADSS tot score (mean ± SD)	3.55 ± 1.25 (*n* = 56)	4.79 ± 2.46 (*n* = 29)	3.15 ± 0.42 (*n* = 40)	< .001	0.234	< .001	< .001
Post-scan RSDI dep/der (mean ± SD)	3.49 ± 1.22 (*n* = 50)	4.84 ± 1.74 (*n* = 29)	2.68 ± 0.44 (*n* = 40)	< .001	< .001	< .001	< .001

*Abbreviations*: *BDI* Beck Depression Inventory, *CADSS* Clinician Administered Dissociative States Scale, *CAPS* Clinical Administered PTSD Scale, *CTQ* Childhood Trauma Questionnaire, *DEP/DER* depersonalization/derealization, *MDI* Multiscale Dissociation Inventory, *PTSD* post-traumatic stress disorder, *PTSD* + *DS* PTSD dissociative subtype, *RSDI* Responses to Script-Driven Imagery Scale, *SD* standard deviation, *STAI* State-Trait Anxiety Inventory

PTSD diagnosis was confirmed through administration of the Clinician Administered PTSD scale-IV (cutoff ≥ 50; [12] or the CAPS-5 [123] (see Table 1 for details). As per standard methods, inclusion in the dissociative subtype of PTSD group (PTSD + DS) required a severity ≥ 4 (frequency + intensity) for CAPS-4, or ≥ 2 (severity) for CAPS-5 on the CAPS depersonalization or derealization symptoms [84, 107, 110, 124. Psychiatric comorbidities were assessed using the Structured Clinical Interview for DSM-IV Axis I Disorders (SCID-I) [37], and additional psychological questionnaires were administered to investigate childhood trauma using the Childhood Trauma Questionnaire (CTQ) [11], dissociative symptomatology using the Multiscale Dissociation Inventory (MDI) [19], and depression symptoms using the Beck Depression Inventory (BDI) [9]. Additional measures were administered at the end of the imaging session to evaluate state anxiety (using the State-Trait Anxiety Inventory (STAI); [101] and state dissociative symptoms (using the Clinician Administered Dissociative States Scale (CADSS) by [17], and the Responses to Script-Driven Imagery Scale (RSDI) by [49] present during the scanning session.

Exclusion criteria comprised history of head injury involving loss of consciousness, current or past history of neurological disorder, history or current psychosis, bipolar disorder, substance or alcohol use disorder, MRI incompatibility, and other relevant medical conditions. Participants included in the control sample did not meet criteria for current or past history of psychiatric disorders, as assessed by the SCID-I and CAPS.

### *MRI Data Acquisition*

Resting-state imaging data were acquired via a whole-body 3 Tesla MRI scanner (Siemens Medical Solutions, Erlangen, Germany) either at the Lawson Health Research Institute for Imaging (Magnetom Verio/Biograph mMR; *n* = 46) or at the Robarts Research Institute for Functional and Metabolic Mapping (Magnetom Trio/Magnetom Prisma; *n* = 87) in London (ON) with the manufacturer’s 32-channel phased array head coil (please refer to Supplementary Materials for the specifics on what scanner model was utilized for each individual per group). Foam pads held the participants’ heads in position. T1-weighted anatomical images were collected using a magnetization-prepared rapid acquisition gradient echo with 1-mm isotropic resolution (TR/TE/TI = 2300 ms/2.98 ms/900 ms, FA 9°, FOV = 256 mm × 240 mm × 192 mm, acceleration factor = 4, total acquisition time = 192 s; FOV = field of view; TR = repetition time; TE = echo time; FA = flip angle).

Next, T2*-weighted functional images were collected with a standard gradient echo planar imaging (EPI) pulse sequence using 2-mm isotropic resolution. EPI volumes were acquired using the following parameters: FOV = 192 mm × 192 mm × 128 mm (94 × 94 matrix, 64 slices), TR/TE = 3,000 ms/20 ms, flip angle = 90°, for a total of 120 volumes.

As per standard methods, participants’ instructions during the 6-min resting-state acquisition were to let the mind wander while keeping their eyes closed [15, 38].

### Data Analyses

#### Demographic and Clinical Measures

We performed Kruskal–Wallis *H* tests, followed by post hoc Mann–Whitney tests, to investigate significant between-group differences regarding age, CAPS, CTQ, MDI, BDI, CADSS, RSDI, and STAI scores. Sex differences were examined using Pearson’s chi-square tests between groups.

#### fMRI Data Preprocessing

fMRI analyses were conducted using SPM12 (Wellcome Trust Center for Neuroimaging, London, UK) and the additional spatially unbiased infratentorial template (SUIT) toolbox (version, 3.1) was implemented in Matlab R2018b (Mathworks Inc., MA) for the investigation of the brainstem and the cerebellum.

Initially, individual functional images were visually inspected to ensure that a complete acquisition of the lower structures of the cerebellum was performed during the scan. A total of 40 subjects were excluded, resulting in 44 healthy controls, 57 PTSD, and 32 PTSD + DS being included in the analyses.

##### Whole-Brain Analysis

For each participant, the preprocessing pipeline included realignment to the first image, re-slicing to the mean functional image, and co-registration to the anatomical image. Image segmentation (gray and white matter, and cerebro-spinal fluid), spatial normalization to the MNI standard template, and smoothing with a 6-mm full-width at half-maximum (FWHM) Gaussian kernel followed. Additional motion correction was implemented using ART toolbox (Artifact Detection Tool version 2015–10; Gabrieli Lab, McGovern Institute for Brain Research, Cambridge, MA) with 2-mm motion threshold. The obtained motion regressors were added to the six standard movement parameters during the first-level analysis [80]. Finally, smoothed images were bandpass-filtered (0.012–0.1 Hz range) using an in-house code (written by co-author J. Théberge).

##### Brainstem and Cerebellum

With regard to the midbrain, lower brainstem, and cerebellum, the functional and anatomical acquired volumes were normalized voxel-by-voxel to the SUIT template (version 3.1) [28, 30]. First, whole-brain anatomical images were segmented and cropped to retain only the brainstem and cerebellum. The obtained structural images were then normalized to the SUIT template using the SUIT-normalization function. Next, the whole-brain realigned and resliced functional images were normalized to the SUIT template by applying the deformation matrix as per the structural images, then cropped to retain the brainstem and cerebellum only, and resliced to a 1.5 × 1.5 × 1.5 mm^3^ voxel size. Finally, the obtained partial-brain functional images were smoothed to a 4-mm FWHM kernel and bandpass-filtered with a high-pass filter of 0.01 Hz and a low-pass filter of 0.8 Hz [8].

#### Seed-Based Connectivity Analyses

**Fig. 1:**
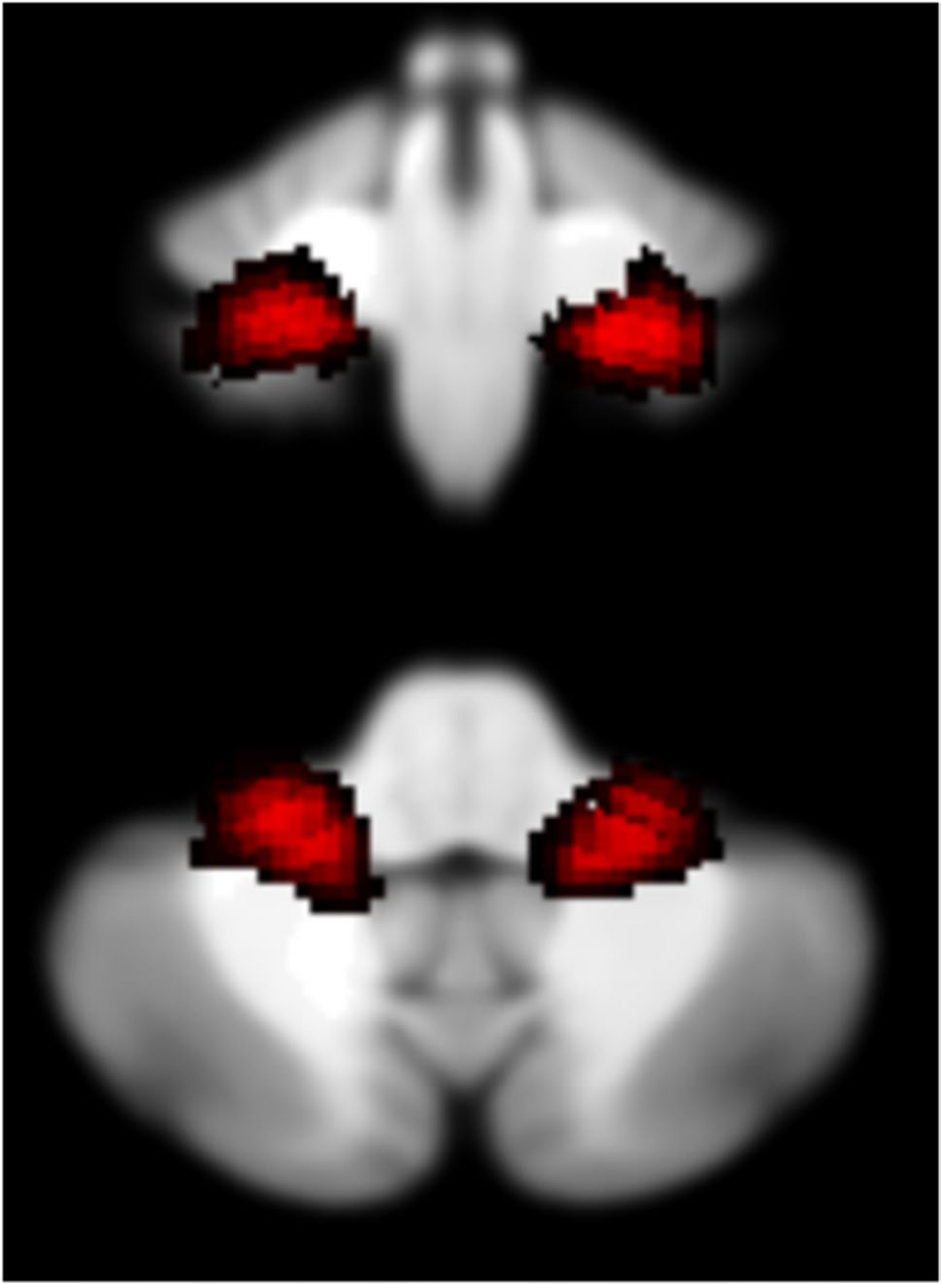
Seed regions displayed on the SUIT space in the coronal (top) and axial (bottom) planes

The seed regions used to perform functional connectivity analyses were generated using SPM Anatomy toolbox [33] as implemented in SPM12 and based upon the cerebellar atlas generated by Diedrichsen et al. [29]. Specifically, distinct left and right flocculus seed regions of interest (ROIs) were created (see Fig. 1).

Each seed regions’ time course mean signal was extracted using SPM Anatomy toolbox and was subsequently input as a regressor in the first-level regression analysis per each subject, in addition to the ART regressor for motion correction. The resulting connectivity reflected a positive correlation between each seed region and any other voxel in the brain. Subsequently, the obtained individual images were used to perform a full-factorial ANOVA that included 3 groups (controls, PTSD, PTSD+DS) × 2 hemispheres (left, right). Post hoc analyses investigated interaction effects between groups for each hemisphere.

Within the SUIT maps, similar to the whole-brain connectivity analyses, each seed region time course mean signal was used as a regressor in the first-level analysis, with additional ART regressors for motion correction. Here, the results yield the functional connectivity between each seed region and each voxel within the partial brain provided by the SUIT template that included the brainstem and cerebellum exclusively. The first-level results were used to perform a full-factorial ANOVA (3 groups, controls, PTSD, and PTSD+DS, × 2 hemispheres, left and right) at the second-level analysis. Within-group analyses (simple *t*-tests using a significant threshold of *p*<.05 FWE-corrected with *k*≥10 at the SUIT map level) were also performed to further explore the resting-state functional connectivity (rsFC) of the flocculus within lower brain structures.

Additional regression analyses were conducted on the entire sample to investigate the association between rsFC of the right and left flocculi and the psychological measures obtained at the assessment, specifically PTSD symptom severity as measured by CAPS total (CAPS-IV and normalized CAPS-5), dissociative symptoms as measured by MDI (using the mean score obtained at the depersonalization and derealization items), childhood trauma history as measured by CTQ, depression as measured by BDI, state dissociation during the scanning session as measured by RSDI (mean scores obtained at the depersonalization and derealization items), and state anxiety during the scanning session as measured by the STAI.

All results were whole-brain corrected at the threshold of α < .05, over an initial uncorrected whole-brain threshold set at *p *< .005 followed by a 1000-iteration Monte Carlo simulation procedure using AlphaSim as implemented in RESTplus Toolbox version 1.8 (http://www.restfmri.net) [99]. This procedure yields minimal cluster extents that ensure a maximum false-positive rate of 5% at the whole-brain level. The individual extent thresholds were calculated per each individual T-map investigated.

An additional a priori region-of-interest analysis was used for the right and left hippocampi, given the role played by these brain structures in PTSD [65, 78, 97, 102], as well as their key role in orienting and spatial navigation [122, 128]. The ROI analysis was implemented on a 10-mm sphere with its peak at MNI ±28, −12, and −24 from Patel et al. [78]. A voxel-wise FWE threshold of *p* < .05 was applied with Bonferroni correction for multiple comparisons, resulting in a local significance threshold of *p *< .025, FWE corrected.

## Results

### Sociodemographic and Psychological Measures

No significant differences were found in age and sex between groups. By contrast, significant between-group differences emerged on all psychological measures, except for CAPS-5 (all healthy controls were assessed with CAPS-IV; see Table 1 for a detailed report).

### rsFC of the Flocculus with the Whole Brain

The full-factorial analysis yielded a significant interaction effect (group by hemisphere) for rsFC between the flocculus and the left precentral gyrus (see Table 2).

**Table 2:** Results of the resting-state functional connectivity analyses of the flocculus. (a) Full factorial results—main and interaction effects, between-group results; (b) regression analysis results

a) Full factorial ANOVA									
		***L/R***	***Brain region***	***k***	***Peak Z***	***Peak p***	***x***	***y***	***z***
**Main effect of group**			ns						
**Main effect of hemisphere**			ns						
**Interaction effect group × hemisphere**		*L*	Precentral gyrus	300	4.54	< 0.001	− 38	2	32
**Between-group results**									
***Seed region***	***Comparison***	***L/R***	***Brain region***	***k***	***Peak Z***	***Peak p***	***x***	***y***	***z***
***Left flocculus***	**HC > PTSD**	*L*	Supramarginal gyrus/TPJ	1210	4.54	< 0.001	− 56	− 38	40
		*L*	Angular gyrus	836	4.48	< 0.001	− 34	− 64	46
		*L*	Superior parietal lobule/IPS/PEF	Subcluster	3.93	< 0.001	− 22	− 62	46
	**PTSD > HC**		ns						
	**HC > PTSD + DS**	*L*	Supramarginal gyrus/TPJ	267	3.94	< 0.001	− 56	− 36	40
		*R*	Precentral gyrus/middle cingulum/PCC	252	3.93	< 0.001	4	− 26	50
		*L*	Angular gyrus/IPS/PEF	621	3.9	< 0.001	− 34	− 62	44
		*L/R*	Medial superior frontal gyrus	281	3.63	< 0.001	2	66	22
		*L/R*	Precuneus	426	3.62	< 0.001	0	− 70	36
	**PTSD + DS > HC**		ns						
	**PTSD + DS > PTSD**		ns						
	**PTSD > PTSD + DS**		ns						
***Right flocculus***	**HC > PTSD**		ns						
	**PTSD > HC**		ns						
	**HC > PTSD + DS**		ns						
	**PTSD + DS > HC**		ns						
	**PTSD + DS > PTSD**	*R*	Hippocampus*	19	3.63	< 0.013*	34	− 8	− 20
	**PTSD > PTSD + DS**		ns						
**b) Correlation with symptoms**									
**CAPS tot**									
***Seed region***	***Correlation***	***L/R***	***Brain region***	***k***	***Z***	***p***	***x***	***y***	***z***
***Left flocculus***	Negative correlation	*L*	Precentral gyrus	4715 (subcluster)	4.42	< 0.001	− 56	12	34
		*L*	Supramarginal gyrus	subcluster	4.24	< 0.001	− 54	− 32	42
		*R*	Supramarginal gyrus	376 (subcluster)	4.24	< 0.001	56	− 36	34
**MDI Dep/Der**	***Correlation***	***L/R***	***Brain region***	***k***	***Z***	***p***	***x***	***y***	***z***
***Right flocculus***	Positive correlation	*R*	Vermis V/VI	331	3.93	< 0.001	0	− 60	− 20
		*L*	Lingual gyrus	244	3.92	< 0.001	− 18	− 68	2
**CTQ tot**	***Correlation***	***L/R***	***Brain region***	***k***	***Z***	***p***	***x***	***y***	***z***
***Left flocculus***	Negative correlation	*R*	Superior frontal gyrus	717	4.58	< 0.001	14	40	42
		*L*	Superior frontal gyrus	197	3.76	< 0.001	− 14	48	34
		*L*	Precentral gyrus	300	3.73	< 0.001	− 58	10	30
		*L*	Superior parietal lobule/angular gyrus	198	3.17	< 0.001	− 28	− 64	40
***Right flocculus***	Positive correlation	*R*	Hippocampus*	27	3.62	< 0.014*	26	− 12	− 18
**RSDI dep/der**	***Correlation***	***L/R***	***Brain region***	***k***	***Z***	***p***	***x***	***y***	***z***
***Left flocculus***	Negative correlation	*R*	Medial superior frontal gyrus	202	4.35	< 0.001	10	40	46
		*L*	Superior parietal lobule	247	3.19	< 0.001	− 22	− 68	46

All results are reported at *p* < .05 whole-brain corrected threshold. * ROI analysis reports *p* < .05 FWE-corrected at the peak level within the ROI. Coordinates are reported in the MNI space

*Abbreviations*: *CAPS* Clinician Administered PTSD Scale, *CTQ* Childhood Trauma Questionnaire, *HC* healthy control group, *IPS* intraparietal sulcus, *k* cluster extent, *L* left hemisphere, *MDI* Multiscale Dissociation Inventory, *ns* not significant results, *PEF* parietal eye field, *PTSD* post-traumatic stress disorder group, *PTSD* + *DS* PTSD dissociative subtype group, *R* right hemisphere, *RSDI* Response Script-Driven Imagery scale, *TPJ* temporo-parietal junction

**Fig. 2:**
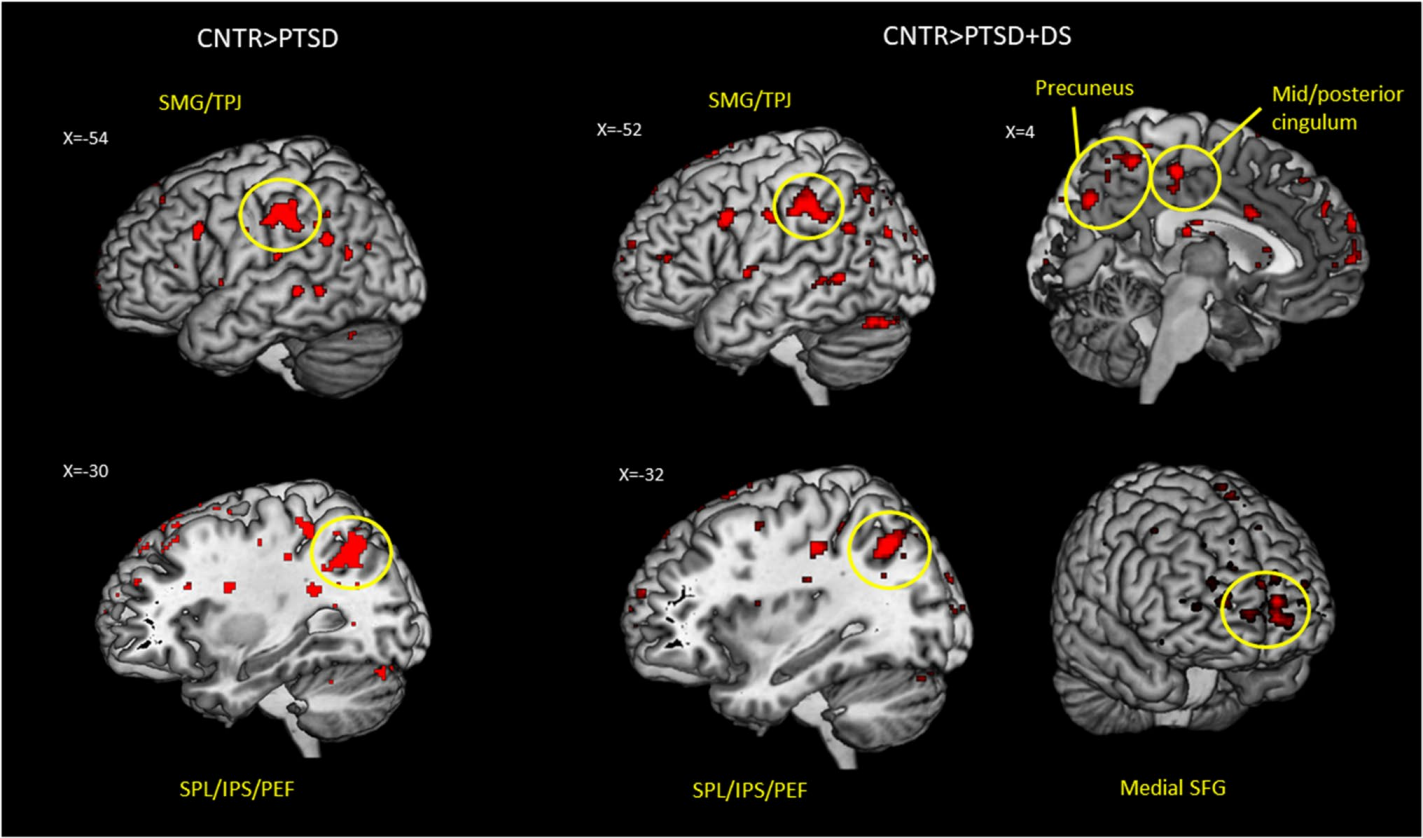
Between-group resting-state functional connectivity results for the *left* flocculus. Decreased rsFC of the PTSD group as compared to controls is shown on the left, decreased rsFC of the PTSD + DS group as compared to controls is shown on the right. Results are shown at a cluster extent threshold ensuring whole-brain correction at a *p* < .05. Abbreviations: CNTR, control group; IPS, intraparietal sulcus; PEF, parietal eye field; PTSD, post-traumatic stress disorder group; PTSD + DS, dissociative subtype of PTSD group; SFG, superior frontal gyrus; SMG, supramarginal gyrus; SPL, superior parietal lobule; TPJ, temporo-parietal junction; > , rsFC greater than

**Fig. 3:**
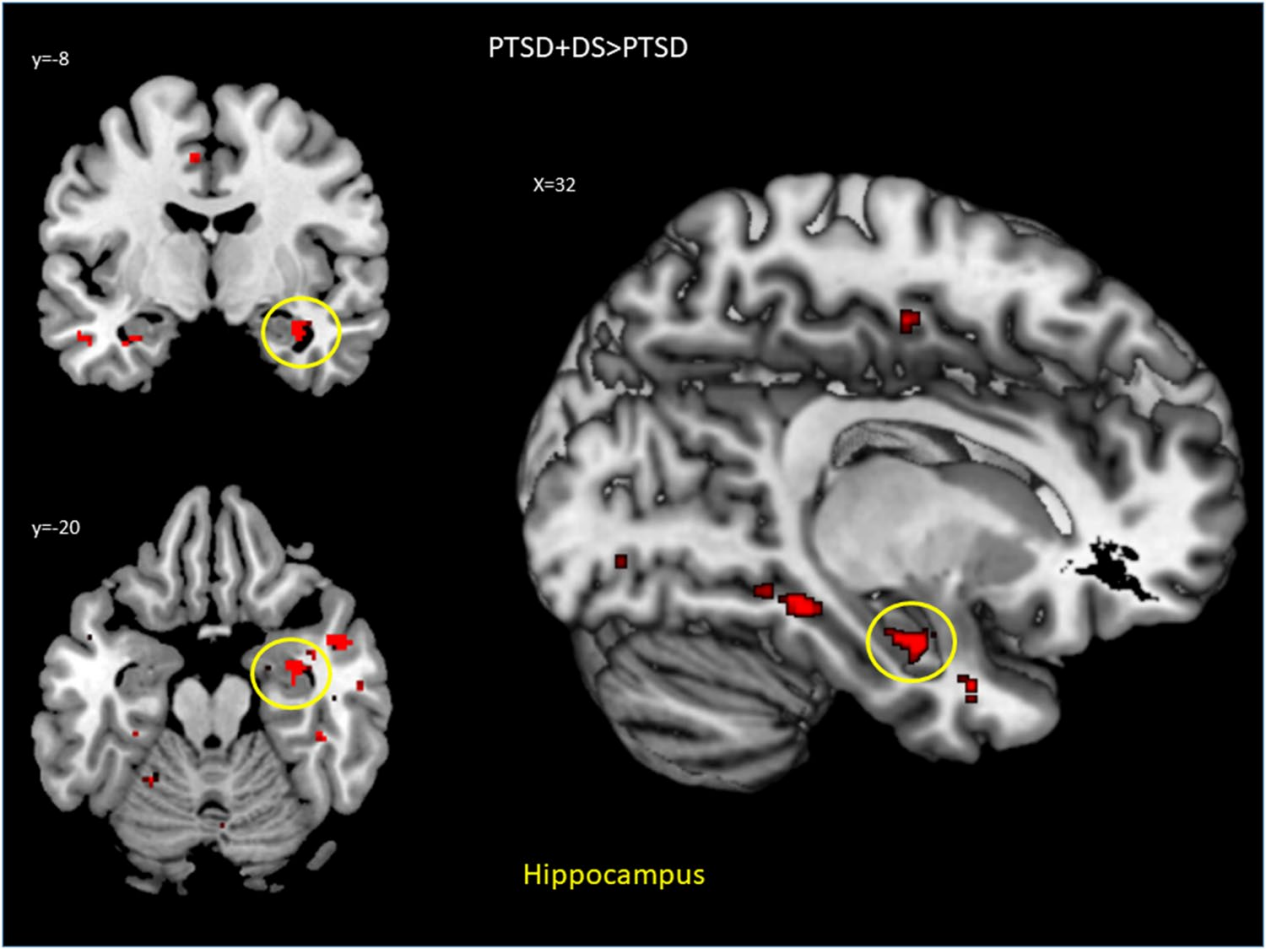
Resting-state functional connectivity results for the *right* flocculus in the PTSD + DS as compared to the PTSD group. Results relative to the hippocampus are shown at a peak *p* < .05 FWE-corrected within the ROI. Abbreviations: PTSD, post-traumatic stress disorder group; PTSD + DS, dissociative subtype of PTSD group; > , rsFC greater than

Post hoc analyses revealed that, as compared to the control group, the PTSD group displayed decreased rsFC of the left flocculus with the left supramarginal gyrus, encompassing the temporo-parietal junction (TPJ), the left superior parietal lobule (including the PEF), and the left angular gyrus. Similarly, as compared to the control group, the PTSD+DS group showed decreased rsFC of the left flocculus with the left supramarginal gyrus, including the TPJ, the right middle/posterior cingulum, the left angular gyrus, the bilateral medial superior frontal gyrus, and the bilateral precuneus. Conversely, the right flocculus showed increased rsFC with the right hippocampus in the PTSD+DS group as compared to the PTSD group (see Table 2 for a detailed report of the results, and Figs. 2 and 3). No other between-group differences emerged.

#### Symptom Correlations with rsFC of the Flocculus

Regression analyses revealed significant correlations between rsFC of the right and left flocculi and several clinical trait and state measures (see Table 2 for an overview of the results). Specifically, PTSD symptom severity, as measured by the CAPS total, was correlated negatively with rsFC of the left flocculus with the left precentral gyrus and with the bilateral supramarginal gyrus. Depersonalization/derealization symptoms, as measured by the MDI, correlated positively with rsFC of the right flocculus with the vermis and the left lingual gyrus. Childhood trauma severity, as measured by the CTQ, correlated negatively with rsFC of the left flocculus with the bilateral superior frontal gyrus, superior parietal lobule and angular gyrus, and the left precentral gyrus. Finally, CTQ scores correlated positively with rsFC of the right flocculus with the right hippocampus.

Finally, state dissociation (depersonalization/derealization as measured by the RSDI) at the time of scanning correlated negatively with rsFC of the left flocculus with the right medial prefrontal cortex and the left superior parietal lobule.

### rsFC of the Flocculus Within the Cerebellum and Brainstem

No main or interaction effects emerged from the full factorial analysis. As a result, no post hoc comparisons were pursued. However, exploratory within-group analyses showed rsFC connectivity of the flocculus within the brainstem and cerebellum for each group separately (see Table S1).

## Discussion

Our results demonstrate important differential resting-state functional connectivity of the flocculus in PTSD, PTSD+DS, and controls. The left flocculus exhibited differential rsFC in PTSD and PTSD+DS as compared to controls, specifically with brain regions involved in the default mode network, including those involved in bodily self-consciousness (supramarginal and angular gyrus, temporo-parietal junction) and medial fronto-parietal regions (precuneus, superior frontal cortex, and mid/posterior cingulum). Interestingly, the only significant difference found between the two PTSD groups centered around increased rsFC of the right flocculus with the ipsilateral hippocampus in PTSD+DS as compared to PTSD.

Finally, functional connectivity within the cerebellum and brainstem regions only (SUIT analysis) did not yield significant between-group results; however, within-group analyses point toward important directions for future research (see Supplementary Materials).

### rsFC of the Flocculus with the Bodily Self-consciousness Network

As compared to controls, both PTSD groups showed decreased rsFC of the left flocculus with a cortical area encompassing the left supramarginal gyrus (SMG) and the TPJ. The SMG and TPJ have been identified as key cortical regions for the development and processing of bodily self-consciousness, an emerging quality of human consciousness that involves self-identification in terms of body-ownership, “this body belongs to me”; self-location, “this is where my body is located”; and first-person perspective, “my body is the place from where I see and interact with the world” [13, 14, 51, 95, 112]. In particular, distortions in self-location provide important foundations in the understanding of depersonalization symptoms in PTSD+DS, where individuals show the capacity to perceive their body as detached from one’s self or not their own [19, 26, 105]. We hypothesize here that a coherent experience of the self in space needs to be embodied in cortical and subcortical structures that support eye, head, and neck movement coordination, along with vestibular function, to orient oneself in space. In line with this hypothesis, in the present study, we observed altered connectivity of the flocculus to the vestibular system in PTSD and PTSD+DS. Previous data have shown altered resting-state functional connectivity of the TPJ/SMG in PTSD+DS as compared to PTSD and healthy controls within the vestibular system [45], where functional connectivity between the vestibular nuclei and the SMG correlated negatively with dissociative symptoms in PTSD at rest [45]. In addition, the left flocculus demonstrated decreased rsFC with the intraparietal sulcus in PTSD and PTSD+DS as compared to controls. The intraparietal sulcus plays a key role in multisensory integration of information regarding the space surrounding the body, namely, the peripersonal space [27, 32, 68]. Here, decreased rsFC with the left flocculus may suggest an impairment in the circuit supporting visuo-spatial processing critical to scanning the environment and gathering visual information within the peripersonal space, with downstream negative consequences on the sense of self in space. Interestingly, rsFC of the left flocculus with the left superior parietal lobule, including the intraparietal sulcus, correlated negatively with CTQ scores, indicating that the higher the severity of PTSD symptoms and childhood trauma, the lesser the rsFC of the left flocculus with cortical regions involved in peripersonal space representation. These correlational results may also have bearing on oculomotion in PTSD and PTSD+DS. The superior parietal region plays an important role in the PEF, where the PEF works in tandem with the frontal eye field and the superior colliculus to produce reflexive saccades, particularly in peripheral vision [72, 79]. Given the role of the flocculus in the production of saccades, our results suggest altered regulation of reflexive saccades in PTSD and PTSD+DS as compared to controls, with dysfunction increasing with the severity of PTSD and childhood trauma.

Interestingly, we also observed decreased functional connectivity of the left flocculus with the angular gyrus at rest in both PTSD and PTSD+DS as compared to controls. Critically, the inferior parietal lobule is a key cortical area for visuo-spatial attention and visuomotor integration [115], with previous research further revealing altered neural activity in the angular gyrus among individuals with depersonalization symptoms [98] and in PTSD [78]. In the present study, rsFC of the left flocculus with the angular gyrus correlated negatively with childhood trauma, suggesting an association of early traumatic experiences with the development of altered brain circuitry involved in spatial awareness and visuomotor integration. Given the central role of these abilities in promoting a coherent sense of self in interaction with the environment, our results are in keeping with the hypothesis that the vestibulocerebellum plays a central role in trauma-related symptomatology relating to bodily self-consciousness.

On balance, our data revealed decreased functional connectivity of the left flocculus with the cortical regions involved in eye and head movement coordination in PTSD and PTSD+DS as compared to controls, which can affect vestibular function, such as balance and gait, and thus impact bodily self-consciousness, including self-location and self-identification.

### rsFC of the Flocculus with the Default Mode Network

Separate consideration is required in examining functional connectivity between the vestibulocerebellum and the default-mode network (DMN), where we have examined already the potential implications of decreased functional connectivity of the flocculus with the TPJ/SMG (cortical regions comprised in the DMN) in both PTSD groups. Notably, the PTSD+DS group also showed decreased functional connectivity between the left flocculus and two key hubs of the DMN, the medial prefrontal cortex, and the precuneus [20, 116, 119]. The DMN appears as the main brain network active during rest in healthy individuals [20, 70]. Critically, neuroimaging research has highlighted aberrant functional connectivity within the DMN in PTSD [2, 3, 15, 55, 59, 85, 113]. Specifically, a significant reduction of functional connectivity within the DMN at rest has been postulated to be associated with a disruption of the sense of self as a consequence of psychological trauma [62]. Here, our results reveal an additional lack of functional connectivity between the DMN and the flocculus in PTSD+DS as compared to controls.

How could oculomotor function relate to the DMN functions? Previous research has shown an association between autobiographical memory retrieval and oculomotion. Here, a study conducted by El Haj et al. [34] assessed eye movements during emotional autobiographical memory retrieval in healthy individuals and observed increased frequency of saccades and fixations, together with decreased fixation duration, in emotional versus neutral memory retrieval. Another study found improvement of episodic autobiographical memory retrieval through horizontal saccadic eye movements [77]. Notably, a well-known therapeutic intervention used in trauma treatment (eye movement desensitization reprocessing (EMDR); [96] successfully facilitates memory retrieval and processing of traumatic memories via the use of saccadic eye movements (for a systematic review, see [57]). Interestingly, a study investigating the neural circuitry underlying autobiographical memory retrieval during a saccadic eye movement task in PTSD as compared to controls revealed aberrant functional connectivity among fronto-parietal regions and cortical areas involved in oculomotion, including the frontal eye field and the supplementary eye field [44]. Taken together, these data suggest an association between eye movements and autobiographical memory retrieval, with the present findings supporting the hypothesis of a disruption in the functional connectivity of the neural circuitry underlying oculomotion and the DMN as a function of psychological trauma, particularly in the dissociative subtype of PTSD.

Notably, the present study also revealed a negative correlation between state depersonalization/derealization symptoms (as measured by the RSDI) and functional connectivity between the left flocculus and the medial prefrontal cortex. These results point toward an association between state dissociation at the time of scanning and disrupted functional connectivity between the vestibulocerebellum and the DMN, likely affecting processing of the sense of self in terms of self-location and self-identification, particularly among individuals with the dissociative subtype of PTSD.

Finally, as compared to controls, PTSD+DS showed decreased rsFC of a cluster encompassing the middle and posterior cingulum. The posterior cingulum plays a central role in autobiographical memory recall [20, 62, 67] and has consistently exhibited altered functional connectivity in PTSD [15, 55, 59, 82, 103]. Interestingly, the posterior cingulum is thought to serve as a key hub for integrating self-location and body ownership representation to build a coherent experience of the bodily self in space [41]. Here, our findings support further the hypothesis that the vestibulocerebellum plays a prominent role in promoting a coherent sense of self in space, including particularly self-location, where dysfunctional rsFC with the posterior cingulum may contribute to compromised bodily self-consciousness in PTSD.

### *Differential rsFC of the Flocculus in PTSD Versus PTSD* + *DS: the Role of the Hippocampus*

Our results reveal increased rsFC of the right flocculus with the ipsilateral anterior hippocampus (mainly CA1 subfield in the uncus) in PTSD+DS as compared to PTSD. In addition, we observed a positive correlation between rsFC of the right flocculus with the anterior hippocampus (comprising CA4 and the dentate gyrus) and childhood trauma.

The hippocampus is thought to play a prominent role in chronic stress and PTSD [18, 35, 64, 73, 108], and high levels of stress hormones, particularly during development, are thought to reduce neurogenesis within the hippocampus, resulting in reduced hippocampal volume in adulthood [1, 16, 18, 35, 54, 108, 109]. 

Notably, the hippocampus has been linked to learning, memory, and visuo-spatial processing facilitating orientation in space [35, 130]. Critically, recent research reveals structural connections between the cerebellum, including the flocculus, and the hippocampus, indicating a direct cerebello-limbic pathway [5]. Here, several indirect pathways support bidirectional functional cerebellar-hippocampal connectivity [128] associated with spatial navigation and time processing tasks in humans and mice models [6, 122, 128].

Our results are therefore consistent with the hypothesis of aberrant hippocampal activity, in this case relative to rsFC with the flocculus, as a consequence of early life trauma. This hypothesis is corroborated by the observation of increased vestibulocerebellum-hippocampal functional connectivity in the dissociative subtype of PTSD, a subtype of PTSD that has been associated with higher levels of childhood trauma, where increased scanning of the environment may be necessary for survival [43, 61, 75, 83, 106, 110].

By contrast, increased flow of stress hormones coupled with the structural and functional alterations characteristic of the hippocampus in PTSD [23, 108] may lead to hyper-connectivity with the vestibulocerebellum necessary to compensate for blunted hippocampal function. The *Cerebellar Universal Transform Hypothesis*, which suggests that the cerebellum plays a critical role in compensating for deficits in brain function [89–91], supports this viewpoint.

Finally, a recent review of the functions of the anterior hippocampus sheds light on our findings, where Zeidmen and Maguire [129] found strong evidence for a role of the medial anterior hippocampus in spatial navigation, referred to as large-scale representation of the external environment [48, 71], especially during recall and imagination of scenes. Here, the hippocampus is thought to aid in linking elements of a scene (recalled or imagined) to produce a coherent spatial representation [129]. In our results, increased FC of the anterior hippocampus with the flocculus at rest may reveal a compensatory attempt to successfully reconstruct a scene representation or search for visuo-spatial inputs to build a more coherent scene in the individual’s mind during self-reflective processing in PTSD+DS. Indeed, depersonalization/derealization symptoms in PTSD+DS involve the perception of one’s body or the environment as unreal, distorted, scattered, and disintegrated, thus suggesting a failed integration of the sensory experience [39, 98, 100].

Here, we propose that depersonalization/derealization symptoms may be linked to disordered sensorimotor integration associated with defective memory recall (scattered recall of images/scene with pieces of sensory information not coherently integrated or misplaced in a scene representation; [10]). The vestibulocerebellum may, in turn, aid the hippocampus during memory retrieval and spatial processing, with heightened scanning of the environment to gather visuo-spatial information or recall of information to reconstruct a scene or an image of one’s self, an ability that appears to be incoherent or disintegrated in individuals with PTSD+DS.

## Limitations

There is a number of limitations to this study. Firstly, the acquisition of data occurred at a single, cross-sectional, point in time; longitudinal studies are therefore warranted. Secondly, neuroimaging data were acquired using a 3-T magnetic field. A higher magnetic field and resolution may help to improve the specificity of the findings, especially in the brainstem regions. Finally, seed-based functional connectivity analysis, although standard in the field, does not allow for an examination of the directionality of functional connectivity between brain regions. No inference can therefore be made with respect to the direction of the impact of one region over another region’s activity.

## Conclusion

Taken together, our results point toward an important role of the left flocculus in differentiating the neural circuits in PTSD and PTSD+DS as compared to controls at rest. In particular, as compared to controls, rsFC of the left flocculus was consistently decreased in relation to several cortical regions involved in bodily self-consciousness, including the TPJ, the SMG, the superior parietal lobule, and the angular gyrus in PTSD and its dissociative subtype. Furthermore, our findings demonstrate decreased rsFC of the flocculus with key regions of the DMN, thus further supporting the notion of aberrant functioning of the default mode network in PTSD and PTSD+DS, with associated consequences for the maintenance of a coherent sense of self. Critically, PTSD+DS appears to be uniquely associated with increased rsFC of the flocculus with the anterior hippocampus, a limbic region strongly affected by early life trauma. These results point toward the need for interventions aimed at increased vestibulocerebellum-hippocampal functional connectivity in the dissociative subtype of PTSD. Furthermore, whereas some PTSD treatments have already integrated psychological interventions using eye movements as a tool for processing traumatic events (EMDR; [96]), our findings suggest the need to further develop bottom-up clinical interventions targeting vestibulo-motor functioning and the integration of multisensory experience (see for example, Deep Brain Reorienting, [25], and Sensory Motor Arousal Regulation Treatment-SMART, [121]) in PTSD and PTSD+DS. We hypothesize that this will facilitate processing of traumatic experiences and help regain optimal cortical functioning, thus restoring the shattered self in the aftermath of trauma.

### Supplementary Information

Below is the link to the electronic supplementary material.

Supplementary file1 (DOCX 23.4 KB)
