# Supplementary material for: The Vestibulocerebellum and the Shattered Self: a Resting-State Functional Connectivity Study in Posttraumatic Stress Disorder and Its Dissociative Subtype
Source: Cerebellum. 2022 Sep 19;22(6):1083–97. doi: 10.1007/s12311-022-01467-4 (PMC10657293; doi:10.1007/s12311-022-01467-4)
Supplement: Supplementary file 1 — Supplementary file1 (DOCX 23.4 KB) [file 12311_2022_1467_MOESM1_ESM.docx]

**1. Results from the exploratory within-group analyses within the SUIT space (brainstem and cerebellum)**

Within the control group, the left flocculus showed significant rsFC with the right lobule VI of the cerebellum, whereas the right flocculus did not yield any significant results.

Within the PTSD group, the left flocculus showed significant rsFC with lobule IX of the right cerebellum and with lobule VIII of the left cerebellum. Moreover, significant rsFC was found between the right flocculus and the right lobules VII and VIII of the cerebellum and between the right flocculus and the left lobule VII, Crus I and II, and Lobule IX of the cerebellum.

Finally, within the PTSD+DS group the left flocculus showed significant rsFC with the left cerebellum, Lobule VII, Crus I, and IX, and with the right lobule VII and VIII of the cerebellum. Similarly, the right flocculus showed significant rsFC with the right lobule VII and VIII and with the left lobule IX of the cerebellum within the PTSD+DS group (see TABLE S1).

Additionally, in the PTSD and PTSD+DS groups, but not in the control group, the left and right flocculi showed significant rsFC with the brainstem nulcei (vestibular nuclei, reticular nuclei, and cranial nerves).

**TABLE S1.** Within-group analysis of the rsFC of the flocculus within the SUIT space (brainstem and cerebellum).

| WITHIN-GROUP COMPARISONS | |  |  |  |  |  |  | **Peak MNI coordinate** |  |
| --- | --- | --- | --- | --- | --- | --- | --- | --- | --- |
| ***GROUP*** | ***Seed Region*** | ***L/R*** | ***Brain region*** | ***k*** | ***Z*** | ***pFWE-corr*** | ***x*** | ***y*** | ***z*** |
| ***Control group*** | **Left flocculus** | *R* | Cerebellum Lobule VI | 57 | 5.42 | <0.001 | 32 | -60 | -25 |
|  | **Right flocculus** |  | ns |  |  |  |  |  |  |
|  |  |  |  |  |  |  |  |  |  |
| ***PTSD group*** | **Left flocculus** | *R* | Cerebellum Lobule IX/X | 1143 | Inf | <0.001 | -22 | -40 | -41 |
|  |  |  | VN/NRTP/PPRF/PN | subcluster | 6.92 | <0.001 | 2 | -32 | -41 |
|  |  |  | VN/NRTP/PPRF | subcluster | 6.52 | <0.001 | -8 | -32 | -41 |
|  |  | *L* | Cerebellum Lobule VIII | 11 | 5.39 | 0.002 | -16 | -68 | -45 |
|  | **Right flocculus** | *R* | Lobule VII/VIII/X | 1431 | Inf | <0.001 | 24 | -40 | -41 |
|  |  |  |  | subcluster | Inf | <0.001 | 36 | -52 | -43 |
|  |  |  |  | subcluster | 7.34 | <0.001 | 46 | -70 | -39 |
|  |  | *R* | Cerebellum Lobule VII Crus II | 19 | 6.15 | <0.001 | 12 | -86 | -39 |
|  |  | *L* | Cerebellum Lobule VII Crus I | 128 | 5.88 | <0.001 | -30 | -68 | -31 |
|  |  | *L* | Cerebellum Lobule VII Crus II | 18 | 5.61 | <0.001 | -36 | -78 | -43 |
|  |  | *L* | Cerebellum Lobule IX | 41 | 5.53 | <0.001 | -12 | -46 | -45 |
|  |  |  |  |  |  |  |  |  |  |
| ***PTSD+DS group*** | **Left flocculus** | *L* | Cerebellum Lobule IX/X | 663 | 7.07 | <0.001 | -20 | -40 | -41 |
|  |  | *R* | NPH/PPRF/PN | subcluster | 6.15 | <0.001 | 14 | -40 | -43 |
|  |  | *L* | Cerebellum Lobule VII Crus I | 11 | 5.3 | 0.001 | -42 | -52 | -39 |
|  | **Right flocculus** | *R* | Cerebellum Lobule VII/VIII/X | 563 | Inf | 0.001 | 20 | -40 | -43 |
|  |  |  | NPH/NRTP/PPRF | subcluster | 6.98 | 0.001 | 6 | -36 | -39 |
|  |  | *L* | Cerebellum Lobule IX | 51 | 5.44 | 0.001 | -22 | -56 | -39 |

*Abbreviations:* NPH: nucleus prepositus hypoglossi; NRTP: nucleus reticularis tegmentis pontis; PN: pontine nuclei; PPRF: paramedian pontine reticular formation; PTSD: post-traumatic stress disorder group; PTSD+DS: dissociative subtype of PTSD group; VN: vestibular nuclei.

**2. Specifics on scanner model per group**

**TABLE S2.** The following table reports how many participants per group were scanned with each MRI scanner model reported. Siemens is the manufacturer for all models.

| **Scanner model** | **Total N** | **Scanner model** | **Controls N** | **Scanner model** | **PTSD+DS N** | **Scanner model** | **PTSD N** |
| --- | --- | --- | --- | --- | --- | --- | --- |
| TrioTim | 69 | TrioTim | 31 | TrioTim | 10 | TrioTim | 28 |
| Prisma fit | 18 | Prisma fit | 13 | Prisma fit | 2 | Prisma fit | 3 |
| Verio | 9 | Verio | 0 | Verio | 8 | Verio | 1 |
| Biograph | 37 | Biograph | 0 | Biograph | 12 | Biograph | 25 |

N = number of participants; PTSD+DS = dissociative subtype of PTSD.

We performed an additional full factorial analysis (3 Groups * 2 Hemisphere) including scanner model as a covariate to control for scanner model as a potential confounding variable. The results were essentially unchanged as compared to the full factorial analysis without scanner model as a covariate.
